# Idelalisib for the Treatment of Chronic Lymphocytic Leukemia

**DOI:** 10.1155/2014/931858

**Published:** 2014-04-01

**Authors:** Maliha Khan, Areeba Saif, Steven Sandler, Aibek E. Mirrakhimov

**Affiliations:** ^1^Department of Internal Medicine, Saint Joseph Hospital, 2900 N. Lake Shore, Chicago, IL 60657, USA; ^2^Dow University of Health Sciences, Karachi 74200, Pakistan; ^3^Department of Internal Medicine, Presence Resurrection Medical Center, 7435 West Talcott Avenue Chicago, IL 60631, USA

## Abstract

Chronic lymphocytic leukemia is the most common leukemia in the United States. It is a slowly progressive disease, with an 82% five-year survival rate. The treatment strategies are highly individualized with patients in the early and stable stages typically not requiring treatment. However, those with progressive or clinically advanced disease will require treatment. Cytotoxic drugs, such as the alkylating agents, purine nucleoside antagonists, and immunotherapeutic agents, have been the mainstay of chemotherapeutic treatment in CLL. However, given the lack of therapeutic specificity, these medications (especially older ones) have limited tolerability due to side effects. In this paper, we will discuss the data on the use of phosphatidylinositol 3 kinase inhibitor Idelalisib in the management of patients with chronic lymphocytic leukemia. The preclinical and clinical data thus far demonstrate that Idelalisib produces a dramatic and durable response in patients with chronic lymphocytic leukemia and without causing significant toxicity. Moving forward, the ongoing clinical trials will help address the various questions currently being raised regarding the long-term application and safety of Idelalisib. With greater clinical experience following more widespread use of Idelalisib, we will be able to determine the optimal combination therapies in treatment-naïve and relapsed/refractory patients, resulting in more individualized therapeutic strategies for patients with chronic lymphocytic leukemia.

## 1. Introduction

Chronic lymphocytic leukemia (CLL) is a lymphoid malignancy characterized by the accumulation and proliferation of nonfunctional and monoclonal small CD5/CD19/CD-20/CD23-positive lymphocytes in the blood, bone marrow, and lymphoid tissues [1, 2]. It is the most common adult leukemia in the United States, with 15,680 new cases and estimated 4,850 deaths reported by the American Cancer Society in 2013 [3]. CLL is primarily a disease of old age with the median age at diagnosis being 72 years; its incidence in the male population is reported to be twice that of the female population [4]. Diagnosis of CLL requires the presence of at least 5,000 monoclonal mature appearing B-lymphocytes per microliter in the peripheral blood [5].

CLL is a slowly progressive disease, with an 82% five-year survival rate [3]. The treatment strategies of CLL are highly individualized with patients in the early and stable stages of CLL not requiring treatment. However, those with progressive or clinically advanced disease will require treatment. Cytotoxic drugs, such as the alkylating agents (chlorambucil, cyclophosphamide, and Bendamustine), have been the mainstay of chemotherapeutic treatment in CLL. However, their lack of specificity for CLL cells and toxicity to normal cells, particularly hematopoietic and immune cells, have limited their efficacy. Other treatment modalities include purine nucleoside analogs (PNA) such as Fludarabine and immunotherapeutic agents such as anti-CD20 monoclonal antibodies (Rituximab, Ofatumumab, and Alemtuzumab) [1, 4, 6]. Several regimens using the combination of immunotherapy with chemotherapeutics drugs are also currently being used in the treatment of CLL. A treatment regimen combining Fludarabine, cyclophosphamide, and Rituximab (FCR) is currently the gold standard of initial treatment for CLL and has also shown response in relapsed/refractory cases [1, 6].

Unfortunately, however, despite the availability of various therapeutic agents for CLL, the disease is currently considered incurable with most patients eventually relapsing after initial treatment. The poor outcomes of the current treatment strategies, especially in patients with high-risk features (del 17p, del 11q, IgVH mutations, ZAP-70, and CD38 expression), and the lack of tolerability of cytotoxic drugs by the older patients have prompted research into the development of novel drug therapies [4, 7]. The standard FCR regimen cannot be tolerated by the majority of CLL patients who begin treatment after the age of 70 and suffer from other comorbid diseases [8]. The advancement in our understanding of the signal transduction pathways involved in CLL has shifted focus towards targeted therapy involving inhibitors of signal transducers in CLL. Some of the drugs being tested in various stages of preclinical and clinical trials include inhibitors of LYN (Dasatinib), SYK (Fostamatinib), PI3K (Idelalisib, Rigosertib), BTK (Ibrutinib, AVL-292), mTOR (Everolimus, Temsirolimus), Cereblon (Lenalidomide), CXCR4/CXCL12 (Nox-A12, Plerixafor), and BCL2 (Navitoclax) [9].

In this review, we particularly focus on the phosphatidylinositol 3 kinase (PI3K*δ*) inhibitor Idelalisib (GS-1101, CAL 101), the clinical rationale behind its use in the treatment of CLL, and the outcomes of various preclinical and clinical trials studying the use of Idelalisib alone or in combination with other therapeutic agents for treatment of both initial and relapse/refractory cases of CLL.

## 2. Biology of PIK3 in CLL

Prior to reviewing the role of PI3K*δ* inhibitor as a therapeutic agent for CLL, it is essential to present a brief overview of the CLL microenvironment and BCR-signaling pathway in B lymphocytes. The intricate interactions between the B cells and their microenvironment are central to the pathogenesis of CLL. CLL cells residing in the body constantly recirculate between the peripheral blood, bone marrow, and the lymphoid organs [7]. While CLL cells residing in the peripheral blood are in a resting state, those located within the bone marrow and secondary lymphatic organs actively proliferate in anatomic tissue sites labelled “proliferation centers” or “pseudofollicles.” Within these proliferation centers, the malignant B cells interact with components of the tissue microenvironment, including bone marrow stromal cells, T cells, and monocyte derived nurse cells [7, 10, 11]. Additionally, there is a complex interplay between B-cell antigen receptor (BCR), chemokines, chemokine receptors, and adhesion molecules, which is responsible for homing, expansion, and survival of the malignant B cells [7, 10].

### 2.1. The B-Cell Antigen Receptor (BCR)

The BCR is transmembrane receptor protein composed of two parts: an antigen-specific membrane bound immunoglobulin (Ig) and an intracellular signal transduction moiety—Ig *α* (Ig*α*, CD79A)/Ig *β* (Ig*β*, CD79B) chains [7, 12]. BCR activation leading to intracellular signaling cascades can either be antigen-induced or may result from an antigen-independent autonomous signaling resulting from interaction between HCDR3 region of the BCR and the FR2 epitope of the same or adjacent BCRs on the same cell [12–14]. BCR stimulation results in the activation of several intracellular signaling pathways, including the PLCy/calcium/NFAT pathway, the PI3K/AKT/mTOR pathway, the NF-*κ*B pathway, and ERK pathway, leading to B-cell maturation and differentiation [10, 14].

### 2.2. PI3K Signaling Pathway in B Cells

PI3K are lipid kinases that play a central role in normal cellular functions such as cell growth, differentiation, proliferation, and survival [15]. Constitutive activation and overexpression of the PI3K pathway have been implicated in the pathogenesis of several human cancers. The PI3K family is subdivided into three classes containing eight different isoforms. The class I kinases, which comprise isoforms PI3K *α*, *β*, *γ*, and *δ*, act by phosphorylation of several membrane inositol phospholipids. Each of the class I PI3K isoforms is heterodimer molecules comprising a catalytic subunit p110 (p110*α*, p110*β*, p110*δ*, or p110*γ*) and a regulatory subunit p85 (p85*α*, p85*β*, p55*γ*, p55*α*, p50*α*, and p101) [16, 17].

Binding of an antigen to the antigen-specific extracellular component of the BCR leads to phosphorylation of ITAMs (immunoreceptor tyrosine-based activation motifs) by tyrosine kinases LYN and spleen tyrosine kinase (SYK). ITAMs are intracytoplasmic specific sequences of the CD79A/B part of BCR which are responsible for further downstream signal cascades in B cells [12]. Upon antigen binding to the BCR, LYN phosphorylates SYK, which in turn amplifies the initial BCR signal by autophosphorylation and phosphorylation of ITAMs [12]. Moreover, LYN also controls inhibition of the BCR signal transduction pathway by activating phosphatases, which inhibit signal transduction through the BCR. LYN and SYK lead to PI3K activation by phosphorylating CD19 and B-cell adaptor protein (BCAP) [18, 19]. This results in recruitment of the p85/p110 complex to the cell membrane. Following activation of PI3K, downstream signaling mediators including AKT, Bruton's tyrosine kinase (BTK), and phospholipase C-*γ* (PLC*γ*2) are recruited [19]. PLC*γ*2 catalyzes the hydrolysis of PIP2 (phosphatidylinositol-4,5-bisphosphate) to release the second messengers DAG (1,2-diacylglycerol) and PIP3 (phosphatidylinositol-3,4, 5-trisphosphate). PIP3 is responsible for the reorganization of the cell cytoskeleton and regulates cellular functions such as cell adhesion via calcium flux into the cells, whereas DAG activates several isoforms of protein kinase C and other downstream signal mediators [19]. The p110*δ* PI3K signaling pathway can also be activated by receptors other than the BCR such as by cytokines IL-4 [16], IL-6, and BAFF [20, 21], by the chemokine CXCL13 [22], and by costimulatory receptors CD40 and Toll-like receptors (TLRs) [23–26].

PI3K*δ* (p110*δ*) is mainly expressed in circulating leukocytes and lymphoid tissues along with the ubiquitously expressed PI3K*α* [23]. It has been demonstrated through various studies in p110*δ* knock-out (KO) and p110*δ* knock-in (KI) mice that p110*δ* is essential for B-cell survival, migration, and proliferation. Mice deficient in a functional p110*δ* protein produced fewer mature B cells and exhibited impaired function in B cells that do manage to develop [12]. Additionally, in CD19/BCAP double knock-out mice, the disruption of PI3K activation arrested generation of immature and mature B cells within the bone marrow and spleen [27]. The importance of p110*δ* is further highlighted by the finding that complete deficiency of PI3K*δ* in cells makes them prone to apoptosis [4], whereas, when a constitutively active form of PI3K*δ* isoform is introduced into cells lacking BCR expression, they are rescued from apoptosis [26]. Furthermore, it has been demonstrated that overexpression of the PI3K*δ* isoform as a wild-type protein induces transformation and constitutive activation of the AKT signaling pathway in cultured cells [4].

The above findings are a clear indication of the importance that the PI3K pathway has not only in normal B-cell development but for the proliferation, survival, and migration of CLL cells.

### 2.3. Targeting PI3K*δ* in B-CLL: Idelalisib (GS 1101; CAL 101)

In CLL tumor cells, there is increased activity of PI3K*δ* in contrast with normal B-cells [17, 18]. The tumor microenvironment, via activation of PI3K*δ*, provides prosurvival signals to CLL cells and also leads to their homing and retention in the bone marrow and lymphoid tissue. Idelalisib (GS-1101 or CAL 101) is a PI3K*δ* isoform specific kinase inhibitor. It has been reported that in vitro Idelalisib, via inactivation of PI3K*δ*, leads to a decrease in AKT and ERK phosphorylation thus interrupting downstream BCR signaling. It induces apoptosis in CLL cells in a time- and dose-dependent manner via a caspase dependent pathway [17, 28]. This cytotoxicity is independent of genomic features such as chromosomal deletions del(11q22.3) and del(17p13.1) and IgVH gene mutational status demonstrating that Idelalisib is equally effective in treating patients with adverse prognostic factors such as p53 deletions and IgVH mutations [4].

Data from preclinical and clinical studies has shown that Idelalisib shows selective cytotoxicity to CLL cells compared with normal B cells. Furthermore, unlike pan-PI3K inhibitors, Idelalisib is not significantly cytotoxic to normal NK and T cells [17]. However, it does alter the function of NK cells and T cells by decreasing production of cytokines IL-6, IL-4, IL-10, and interferon-*γ* by these immune effector cells resulting in impaired CLL cell proliferation [4, 29]. Moreover, Idelalisib antagonizes the microenvironmental triggers TNF-*α*, CD40L, BAFF, and fibronectin, all of which act by increasing AKT phosphorylation in CLL cells and protecting them from spontaneous apoptosis [4, 17, 29].

These findings demonstrate that Idelalisib targets multiple mechanisms of CLL cell survival by not only direct inhibition of the PI3K signaling pathway but also by inhibition of extrinsic activation of this pathway by microenvironmental stimuli from CD40L, BAFF, TNF-*α*, and fibronectin. This provides a strong rationale for the use of Idelalisib as a therapeutic agent in CLL patients.

### 2.4. Clinical Evidence for Use of Idelalisib as a Therapeutic Agent in CLL

Multiple preclinical and clinical studies are being performed to investigate Idelalisib as a therapeutic agent for CLL as monotherapy or in combination with Rituximab and/or Bendamustine, Fludarabine, Ofatumumab, Chlorambucil, Chlorambucil + Rituximab, and Lenalidomide + Rituximab. A study investigating the synergistic effect of Idelalisib in combination with histone deacetylase inhibitor (HDI), panobinostat (LBH589), and suberoylanilide hydroxamic acid (SAHA) in vitro has also been performed in CLL and primary non-Hodgkin lymphoma (NHL) [30]. The study indicated that PI3K inhibitor synergistically potentiates histone deacetylase inhibitor-induced proliferation inhibition and apoptosis in malignant hematopoietic cells through the inactivation of PI3K and extracellular signal-regulated kinase pathways.

The first phase I trial on Idelalisib studied the clinical pharmacokinetics of the drug in healthy volunteers and in those with lymphoid malignancies [31]. Following administration of single doses up to a maximum of 400 mg and multiple doses of up to 200 mg twice a day for a week in healthy subjects, it was found that Idelalisib is well tolerated with no apparent maximum tolerated dose. Subsequently, Idelalisib was tested in patients with lymphoid malignancies in dose levels up to a maximum of 350 mg/kg over several months. The drug was also well tolerated in these patients with reversible transaminase elevations in only some patients with lymphoid malignancies. The clinical data from this study supports dosing at ≥150 mg twice a day as this achieves steady-state plasma concentrations. Idelalisib can be orally administered with or without food and is metabolized to a single metabolite in the plasma with hepatic metabolism. It is interesting to note that, although Idelalisib is metabolized by the CYP450 3A4 enzyme, it does not appear to be a sensitive substrate for the enzyme and can be coadministered with inhibitors of CYP450 3A4.

Preliminary reports from a phase I trial studying the clinical activity of Idelalisib in relapsed or refractory B-cell malignancies such as chronic lymphocytic leukemia (*N* = 55), indolent non-Hodgkin lymphoma (*N* = 63), mantle cell lymphoma (*N* = 40), diffuse large B-cell lymphoma (*N* = 9) as well as in acute myelogenous leukemia (*N* = 12), and multiple myeloma (*N* = 12) has shown that responses were obtained in patients with CLL (4/17), indolent lymphoma (9/15), and mantle cell lymphoma (6/7) at all dose levels [32]. However, no response was visible in patients with acute myeloid leukemia or diffuse large B-cell lymphoma. The level of pAKT expression was measured in some CLL patients and was found to be markedly decreased following Idelalisib dosing on day 1 (1.5 h and 4 h after dosing) and on day 8 prior to dosing.

In the final results reported from a phase I study [33] investigating the clinical activity of Idelalisib in fifty-four patients with relapsed/refractory CLL, being given single-agent oral Idelalisib 50–350 mg/dose, overall response rate (ORR) was 56% including two complete remissions (CR) and twenty-eight partial remissions (PR). Of the twenty-eight PR, twenty-two met the Hallek criteria (2008) [34] and six met the PR with lymphocytosis Cheson criteria (2012) [35]. The median time to first response was 1.9 months while the median progression free survival (PFS) was 17 months. Furthermore, with treatment 81% patients showed a lymph node response that is a ≥50% reduction in the nodal SPD (sum of the product of the longest perpendicular dimensions); there was a resolution of splenomegaly (70% patients) and normalization of cytopenias: anemia (68%), thrombocytopenia (79%), and neutropenia (100%). There were no dose-limiting toxicities reported and the most common adverse effects included fatigue, diarrhea, pyrexia, rash, upper respiratory tract infection, and pneumonia. Of the 15% patients that discontinued due to adverse effects, 7% were potentially treatment-related.

As with other novel agents targeting the BCR pathway in CLL, an initial increase in the absolute lymphocyte count (ALC) is seen with Idelalisib therapy. According to the IWCLL response criteria [34], lymphocytosis is a sign of disease progression; however, Cheson et al. [35] have recommended a modification of the response criteria and cautioned against labelling the peripheral lymphocytosis initially seen with BCR-targeting drugs as a progressive disease. This lymphocytosis may be attributed to reduced CLL cell adhesion to stromal cells and reduced responsiveness to CXCL12 and CXCL13, which may interfere with CLL cell tissue homing. Consequently, in the peripheral blood the lack of prosurvival signals from the tissue microenvironment makes the CLL cells prone to programmed cell death or apoptosis [7]. One of the approaches to eliminate the increase in ALC to keep with the current response criteria has been to study Idelalisib in combination with other therapeutic agents. Preliminary results from a phase I study indicate that combinations of Idelalisib with Fludarabine, Bendamustine, and/or Rituximab resolve the peripheral lymphocytosis seen with Idelalisib monotherapy [4].

In a recent study investigating combinations of Idelalisib with Rituximab and/or Bendamustine, Coutre et al. report that when Idelalisib (150 mg BID continuously) was given with Rituximab at a dose of 375 mg/m^2^ weekly for 8 doses and/or 90 mg/m^2^ Bendamustine on days 1 and 2 of each cycle for 6 cycles, overall response rates (ORR) for the Idelalisib + Rituximab, Idelalisib + Bendamustine, and Idelalisib + Rituximab + Bendamustine regimens were 78%, 82%, and 87%, respectively, and the one-year progression-free survival (PFS) rates were 74%, 88%, and 87%, respectively [36]. Moreover, almost all studied patients showed a rapid decrease in lymphadenopathy. Some of the Grade ≥3 adverse events were anemia, neutropenia, febrile neutropenia, thrombocytopenia, infections, pneumonia/pneumonitis, rash, diarrhea, and hepatic transaminase elevation. In a phase I study, Barrientos et al. [37] studied Idelalisib in combination with Rituximab and Bendamustine in patients with relapsed or refractory CLL. When Idelalisib was given with Rituximab and/or Bendamustine in the identical dosing regimens specified above, overall response rates (ORR) were reported to be 81%, with 1 complete remission (CR). The median time to response was 1.9 months; the 2-year progression-free survival (PFS) was 62% and OS (overall survival) was 85%. The AEs (any Grade/≥Grade 3) were pyrexia, diarrhea, cough, fatigue, nausea, pneumonia, rash, and ALT/AST elevation. In another phase I study evaluating the combination of Idelalisib with Bendamustine/Rituximab or Chlorambucil/Rituximab in relapsed/refractory CLL patients, Wagner-Johnston et al. reported an ORR of 89.7%, demonstrating good efficacy and tolerability of Idelalisib in these patients when used in combination regimens [38]. Vos et al. studied the combination of Idelalisib with Bendamustine, Fludarabine, or Chlorambucil in patients with relapsed or refractory CLL in a phase I study and reported an ORR of 78% and estimated DOR and PFS of 28.5 months [39]. The results from these studies indicate that double or triple combination treatment regimens of Idelalisib with Rituximab, Bendamustine, Chlorambucil, or Fludarabine are highly active with an excellent safety profile and tolerability and necessitate further phase III studies evaluating their efficacy and safety. A phase III trial evaluating the efficacy and safety of Idelalisib in combination with Bendamustine and Rituximab for previously treated CLL was initiated in June, 2012, and is currently underway [40].

A phase I study evaluating Idelalisib in combination with Rituximab or Ofatumumab was conducted in patients with relapsed/refractory CLL [41]. They were given Idelalisib continuously at 150 mg BID in combination with a total of 8 infusions of Rituximab (R, 375 mg/m_2_ weekly × 8) or a total of 12 infusions of Ofatumumab (O, 300 mg initial dose either on day 1 or day 2 relative to the first dose of Idelalisib, then 1,000 mg weekly for 7 cycles, and then 1,000 mg every 4 weeks for cycles). Those patients who were on treatment after 48 weeks were eligible to continue Idelalisib on an extension study. The ORR was 83% with 3 CRs (8%) and a median time to response of 1.9 months. The PFS for all patients and duration of response (DOR) were 20 and 19 months, respectively. Among the 11 patients with del(17p) and/or TP53 mutations, the response rate was 73% and the median PFS and DOR were 19 months. This study demonstrates that the combination of Idelalisib with anti-CD20 antibodies such as Rituximab or Ofatumumab has good clinical activity and tumor control along with an acceptable safety profile in patients with relapsed/refractory CLL. Phase III trials evaluating the efficacy and safety of Idelalisib in combination with these two anti-CD20 antibodies, Rituximab or Ofatumumab, are currently underway (NCT01539512, NCT01659021) [42].

In a phase II study, O'Brien at al. investigated the combination of Idelalisib with Rituximab in treatment-naive patients ≥65 years of age, with CLL or SLL [43]. They were treated with Rituximab 375 mg/m^2^ weekly × 8 doses and Idelalisib 150 mg BID continuously for 48 weeks; those who completed 48 weeks without progression were enrolled in an extension study and continued to receive Idelalisib. The ORR was 96%, and the median time to response was 1.9 months with no on-study relapses. At 24 months, the estimated PFS was 91%. Most notably, all of the six patients with 17p deletion (del(17p)) responded with 1 CR and 5 PR. The AEs reported were diarrhea, pyrexia, chills, fatigue, rash, pneumonia, and elevated ALT/AST.

There are several genetic mutations that serve as markers of poor prognosis in CLL including del(17p)/TP53 mutation, del(11q), IGHV mutation, and NOTCH1 mutation [44–46]. Coutre et al. reported the effect of these mutations on the clinical activity of Idelalisib in both treatment-naïve and relapsed/refractory CLL patients [47]. All subjects were enrolled into one of three trials, the first two of which were for relapsed/refractory patients; a phase I study with monotherapy, escalation dosing from 50–350 mg BID; phase I combination with either Rituximab, Ofatumumab, Bendamustine ± Rituximab, Fludarabine, or Chlorambucil ± Rituximab; phase II combination with Rituximab in previously untreated patients aged ≥65 years. The results from this study indicate that Idelalisib shows good activity in CLL regardless of these high-risk prognostic markers.

A combination of Idelalisib with CX-4945, an orally bioavailable selective inhibitor of Casein Kinase 2 (CK2), has also been tested and has shown promising activity in CLL [48]. Another preclinical study exploring the synergism between Idelalisib and a highly selective spleen tyrosine kinase (Syk) inhibitor, GS-9973, has shown significant activity of this combination in samples collected from CLL patients [49]. Preclinical studies also support a combination of Lenalidomide with Idelalisib. Lenalidomide-mediated disease-specific toxicity of tumor flare and cytokine release is attenuated by inhibition of PI3K pathway by Idelalisib [50].

Furman et al. enrolled 220 patients with relapsed CLL and coexistent medical conditions such as decreased renal function, chemotherapy related bone marrow suppression, and other major comorbidities [51]. The patients were divided into two groups: Rituximab with Idelalisib and Rituximab with placebo. It was demonstrated that combination with Rituximab and Idelalisib led to greater disease free survival, treatment response rate, and survival among patients with relapsed CLL.

### 2.5. The Future Perspective: Long-Term Application of Idelalisib

To date, the results from trials evaluating the clinical efficacy and safety of Idelalisib, both as monotherapy and as part of combination regimens, have been encouraging. However, there are still questions to be answered about the long-term application of Idelalisib, particularly about its safety, tolerability, drug interactions, and most importantly the duration and durability of response. There is a concern that, with specific inhibition of such a complex pathway as the PI3K pathway, there exists a potential for escape by upregulation or activation of other PI3K isoforms, resulting in drug resistance [50]. Future research into Idelalisib therapy for CLL will have to focus on all these aspects including determination of the optimal combination regimens and long-term outcomes in both previously untreated and refractory/relapsed CLL patients.

## 3. Conclusion

The extraordinarily promising results shown by targeted therapy have revolutionized the current treatment strategies for CLL. The preclinical and clinical data thus far demonstrate that Idelalisib produces a dramatic and durable response in CLL patients without causing significant toxicity, which is a drawback of other nonspecific therapies. Moving forward, the ongoing clinical trials will help address the various questions currently being raised regarding the long-term application and safety of Idelalisib. With greater clinical experience following more widespread use of Idelalisib, we will be able to determine the optimal combination therapies in treatment-naïve and relapsed/refractory patients, resulting in more individualized therapeutic strategies for CLL patients.
